# Potassium Chloride, Sodium Lactate and Sodium Citrate Impaired the Antimicrobial Resistance and Virulence of *Pseudomonas aeruginosa* NT06 Isolated from Fish

**DOI:** 10.3390/molecules28186654

**Published:** 2023-09-16

**Authors:** Natalia Tomaś, Kamila Myszka, Łukasz Wolko

**Affiliations:** 1 Department of Biotechnology and Food Microbiology, Poznan University of Life Sciences, Wojska Polskiego 48, 60-637 Poznań, Poland; 2 Department of Biochemistry and Biotechnology, Poznan University of Life Sciences, Dojazd 11, 60-632 Poznań, Poland; lukasz.wolko@up.poznan.pl

**Keywords:** minimally processed fish, antibiotic resistance, efflux pump, foodborne pathogen, alginate, RNA-seq, RT–qPCR

## Abstract

Sodium chloride (NaCl) is a commonly used additive in minimally processed fish-based products. The addition of NaCl to fish products and packaging in a modified atmosphere is usually efficient with regard to limiting the occurrence of the aquatic environmental pathogen *Pseudomonas aeruginosa.* Given the negative effects of excess NaCl in the diet, there is a growing demand to reduce NaCl in food products with safer substituents, but the knowledge of their impact on antibiotic resistant *P. aeruginosa* is limited. This study aimed to evaluate the physiological and transcriptome characteristics of *P. aeruginosa* NT06 isolated from fish and to determine the effect of selected concentrations of alternative NaCl compounds (KCl/NaL/NaC) on the *P. aeruginosa* NT06 virulence phenotype and genotype. In the study, among the isolated microorganisms, *P. aeruginosa* NT06 showed the highest antibiotic resistance (to ampicillin, ceftriaxone, nalidixic acid, and norfloxacin) and the ability to grow at 4 °C. The Comprehensive Antibiotic Resistance Database (CARD) and the Virulence Factor Database (VFDB) revealed the presence of 24 and 134 gene products assigned to AMR and VF in the *P. aeruginosa* NT06 transcriptome, respectively. KCl, KCl/NaL and KCl/NaL/NaC inhibited pyocyanin biosynthesis, elastase activity, and protease activity from 40 to 77%. The above virulence phenotypic observations were confirmed via RT–qPCR analyses, which showed that all tested AMR and VF genes were the most downregulated due to KCl/NaL/NaC treatment. In conclusion, this study provides insight into the potential AMR and VF among foodborne *P. aeruginosa* and the possible impairment of those features by KCl, NaL, and NaC, which exert synergistic effects and can be used in minimally processed fish-based products.

## 1. Introduction

The addition of sodium chloride (NaCl) is an effective and commonly used method for preservation of minimally processed seafood. The ability of NaCl addition to reduce spoilage and the growth of pathogenic bacteria in seafood is mainly attributed to lowered water activity. Moreover, NaCl causes osmotic stress toward microorganisms and decreases oxygen solubility, which are major bacterial growth limitation factors [1]. The preservative effects of NaCl are essential for ensuring the shelf life and safety of minimally processed ready-to-eat (RTE) fish-based products, which usually receive no heat treatment during processing [2]. The food market of these products is constantly growing, due to both an increase in aquaculture production and consumer demand [3]. At the same time, because the excess NaCl intake in the diet (according to The World Health Organization, more than 5 g of NaCl per day) results in hypertension, the development of cardiovascular diseases, gastric cancers and obesity, the current recommendations for reducing the amount of NaCl within the product have recently been of great importance [4]. Therefore, minimally processed fish-based products, for which NaCl is still an important preservative agent, should be preserved with alternative compounds or techniques [5]. Hence, there is an urgent need to conduct research on the impact of alternative NaCl compounds on specific spoilage and pathogenic bacteria related to aquatic environments. 

The possible NaCl alternatives that can be implemented in fish-based products are potassium chloride (KCl), sodium lactate (NaL), and sodium citrate (NaC) [6]. Research concerning partial NaCl replacement with those substituents is now gaining much attention in food microbiology. For instance, replacement NaCl with KCl and calcium ascorbate resulted in decreased microbial contamination and improved bacon overall quality [7]. The impact of 25% and 50% NaCl replacement on smoked salmon sensory and microbiological properties has also been demonstrated by Muñoz and coauthors [8]. NaCl substituents, e.g., KCl, CaCl_2_, and MgCl_2_, added to meat and seafood exhibited equivalent ionic strengths and did not result in differences in oxidation stability [9]. Nevertheless, as most of the studies were focused on sensory and organoleptic changes upon NaCl substitution, few of them considered the impact on the physiology of microorganisms present in foods [5].

*Pseudomonas* spp. are one of the main groups of bacteria responsible for fish spoilage, which manifests as changes in taste, smell and appearance [10,11]. Among pseudomonads, the major threat both to food quality and safety is *P. aeruginosa* [12]. *P. aeruginosa* is an opportunistic pathogen that causes acute and chronic infections due to its capacity to produce a large repertoire of virulence factors (VF) that are regulated by the *quorum sensing* system [13]. Recent studies revealed the presence of VF among pseudomonads obtained from clinical isolates [14,15,16,17], while little is known about the physiological characteristics of pathogenic foodborne strains. Notably, *P. aeruginosa* prevalence among fish and minimally processed fish-based products has constantly increased. *P. aeruginosa* cells were present in 31.57% of fresh fish collected from aquaculture farms [18]. Similarly, 33.1% and 20% of fresh and smoked fish, respectively, contained *P. aeruginosa* cells [19]. A recent study also found that in 470 fish samples, *P. aeruginosa* strains were present in 14.7% of fresh fish, 4% of dried fish, and 2.85% of salted and smoked fish [12]. In addition, pseudomonads isolated from food were capable of producing a wide range of pigments and forming biofilms [20,21]. Pyocyanin produced by almost all *P. aeruginosa* strains is a blue pigment that belongs to phenazines, and its synthesis enhances cell persistence [22]. Elastase and protease encoded by the lasB and aprA genes, respectively, are extracellular enzymes that induce tissue damage through degradation processes [23]. *P. aeruginosa* isolated from meat and carcasses contained a number of VF genes, among which exoS, algD, lasA, plcH, and exoU were most frequently detected [24]. 

*P. aeruginosa* isolated from seafood is also burdened with a constantly expanding antimicrobial resistance (AMR) phenotype and genotype [25,26,27]. Consequently, an increasing number of outbreaks caused by *P. aeruginosa* isolated from such products may occur [28,29]. Furthermore, since the vast majority of fish on the food market originate from aquaculture, where in general, there is a need for antimicrobials, AMR among fish-related bacteria can also be a problem [29,30]. Moreover, food-associated *P. aeruginosa* is able to rapidly develop resistance to a wide range of antimicrobials [31,32]. The prevalence of AMR *Pseudomonas* spp. was also shown in the diary sector, where resistance to β-lactams was the most prominent [21]. *P. aeruginosa* isolated from fresh fish samples was resistant to amoxicillin, cefotaxime, tetracycline, and gentamicin [18]. The antibiotic resistance profile of *Pseudomonas* obtained from a salmon processing environment concerned ampicillin and amoxicillin [29]. AMR phenotypes of *Pseudomonas* spp. isolated from fresh fish fillets included resistance to penicillin, ampicillin, amoxicillin, and tetracycline [33]. A number of studies have demonstrated that *P. aeruginosa* enhanced resistance to antimicrobials and virulence is due to the genome-encoded AMR systems, e.g., efflux pumps, antibiotic inactivation enzymes, and two-component regulatory systems, indicating increasing concern about foodborne AMR bacteria [16,34,35,36].

The correlation between food-associated stress factors and bacterial resistance has been comprehensively reviewed in the recent work of Liao and coauthors [37]. However, detailed knowledge of *P. aeruginosa* prevalence in fish and the impact of alternative preservative antimicrobial compounds is limited. Therefore, precise examination of the physiology of *P. aeruginosa* foodborne isolates in model experiments and their potential antibiotic resistance are important factors for maintaining high food quality and safety. The aim of the present study was to characterize the antimicrobial and virulence potential of fish-derived *P. aeruginosa* and to evaluate the impact of selected concentrations of KCl/NaL/NaC on AMR and VF via *(i)* the determination of the antibiotic resistance among *P. aeruginosa* strains by the disc diffusion method; *(ii)* identification of AMR- and VF-related genes in the most resistant *P. aeruginosa* fish isolate; *(iii)* evaluation of the impact of selected concentrations of NaCl alternative compounds on the *P. aeruginosa* virulence phenotype (i.e., pyocyanin content, elastase and protease activities); and *(iv)* assessment of the expression levels of genes involved in AMR and VF upon treatment with selected concentrations of NaCl alternative compounds in vitro and in situ.

## 2. Results

### 2.1. Antimicrobial Resistance among P. aeruginosa Strains

The resistance profile of the six *P. aeruginosa* strains isolated from commercially available salmon was phenotypically characterized by the ability to grow under refrigerated temperature and by using eight antibiotics that represent a different class of antibiotics, as shown in Table 1. All analyzed strains were able to grow at refrigerated temperatures, but only strain NT06 grew effectively after just 24 h. Of the eight tested antibiotics, ampicillin (AMP), ceftriaxone (CRO), and nalidixic acid (NA) showed no bactericidal effect on the examined *P. aeruginosa* strains. There were no significant (*p* < 0.05) differences in sensitivity to tetracycline (TE) among *P. aeruginosa* strains; the zones of inhibition were approx. 12 mm, indicating an intermediate resistance pattern [38]. All *P. aeruginosa* strains were sensitive to gentamicin (GEN), meropenem (MEM), and ciprofloxacin (CIP), while norfloxacin (NOR) was not effective against *P. aeruginosa* NT06. 

### 2.2. P. aeruginosa NT06 Genes Involved in AMR and VF

Based on its ability to grow at low temperatures and its antibiotic resistance profile, only *P. aeruginosa* NT06 was chosen for transcriptome analysis. The strain was sequenced, and the obtained data were deposited as NCBI RNA-Seq data in the NCBI Short Read Archive (SRA) under the number SRX19555927. Screening of the CARD and VFDB databases resulted in a total of 24 and 134 genes assigned to AMR and VF, respectively (Table 2 and Table 3). Among the AMR genes in the *P. aeruginosa* NT06 transcriptome, those related to the “efflux pump complex or subunit conferring antibiotic resistance” predominated. For example, homologues of components of the MexAB-OprM efflux pump and multidrug outer membrane proteins, i.e., OpmH, OprN, and OpmE and membrane fusion proteins, i.e., MexP, MexJ, MexX, MexL, and TriA, were present. In the *P. aeruginosa* NT06 transcriptome, five genes with at least 98.94% homology with genes encoding proteins and two-component regulatory systems modulating antibiotic efflux, rsmA, rrmR, armR, parS, basS, and mexL, were also detected. Moreover, four members of “antibiotic inactivation enzymes” (OXA-850, OXA-486, fosA, and APH(3’)-IIb) and one gene-altering cell wall charge (arnA) were found. 

The major VFs identified in the *P. aeruginosa* NT06 transcriptome were classified as flagella and type IV pili, which accounted for 25 and 24 genes, respectively. Among the flagella group, the genes implicated in flagellar structure and biosynthesis proteins were present, while twitching motility, fimbrial biogenesis, and chemotaxis proteins were the examples of the type IV pili group. Additionally, members of alginate biosynthesis and regulation (algU-mucA-mucB-mucC-mucD and algR-algZ) and rhamnolipid (rhlA, rhlB, rhlC) were identified. Other VFs in the *P. aeruginosa* NT06 transcriptome included secretion systems, as follows: HSl-1 (secretion island I), TTSS (type III), and xcp (type II) secretion systems, as well as genes encoding siderophores, i.e., phenazines, pyochelin, pyocyanin, and pyoverdine. Finally, quorum sensing-related genes (lasI, rhlI, aprA, lasA, and lasB) were also present. 

However, not all genes identified in the de novo assembled transcriptome genes were also identified in the mapped transcriptome; thus, the abundance values were not calculated. The abundance of each identified gene transcript was normalized with a TPM value that indicated the number of transcripts that came from 1 million RNA molecules. The range of TPM values was between 8.41 and 110.98 in the case of CARD-identified genes, and from 0.25 to 419.40 in the group of VF genes. 

### 2.3. The Effects of NaCl Alternatives on the P. aeruginosa NT06 Virulence Phenotype

**Figure 1:**
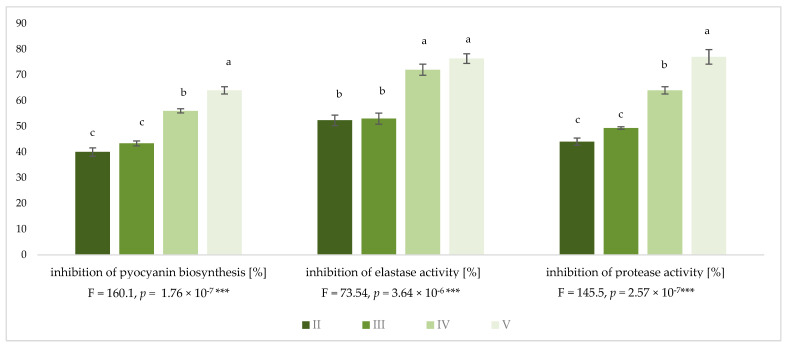
Impact of alternative NaCl compounds on the *P. aeruginosa* NT06 virulence phenotype. Values are the average percentage of inhibition of test activity relative to control cultures. Means with the same letter do not differ significantly. “***” indicates significance level of *p* < 0.001. Medium variants: II—5.0 g/L KCl; III—6.0 g/L KCl; IV—KCl 6.0 g/L + NaL 6.0 g/L; V—KCl 6.0 g/L + NaL 6.0 g/L + NaC 2.5 g/L.

To investigate the impact of KCl, NaL and NaC on the *P. aeruginosa* NT06 virulence phenotype grown on mTSB medium, spectrophotometric analyses determining the pyocyanin biosynthesis, and elastase and protease activities were performed. The results are presented in Figure 1, as the percentage of inhibition with regard to the control culture (mTSB medium supplemented with 5 g/L NaCl). 

There were no significant differences in virulence phenotype inhibition in the case of KCl treatment at both tested concentrations (5 and 6 g/L); the pyocyanin biosynthesis, elastase and protease activity were inhibited by approximately 40%, 52% and 45%, respectively. The addition of NaL to KCl resulted in a considerably higher reduction in pyocyanin biosynthesis (an average of 56%) and protease activity (an average of 64%), while the combination of KCl/NaL/NaC was the most effective at retarding the *P. aeruginosa* NT06 virulence phenotype. The above treatment decreased pyocyanin biosynthesis and protease activity by 64% and 77%, respectively. No significant changes in elastase activity were observed for KCl/NaL and KCl/NaL/NaC treatments, which resulted in 72 and 76% elastase inhibition, respectively. 

### 2.4. The Effect of NaCl Alternatives on Genes Involved in AMR and VF

**Figure 2:**
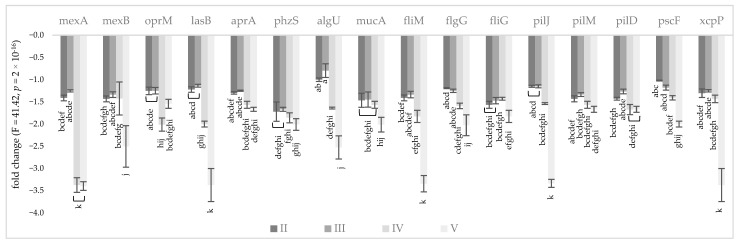
Impact of alternative NaCl compounds on the *P. aeruginosa* NT06 AMR and VF gene expression levels under in vitro conditions. Values are presented as a fold change in expression in relation to control cultures and normalized to the non-differentially expressed 16S rRNA gene. Error bars indicate standard deviations from three replicates. Medium variants: II—5.0 g/L KCl; III—6.0 g/L KCl; IV—KCl 6.0 g/L + NaL 6.0 g/L; V—KCl 6.0 g/L + NaL 6.0 g/L + NaC 2.5 g/L.

**Figure 3:**
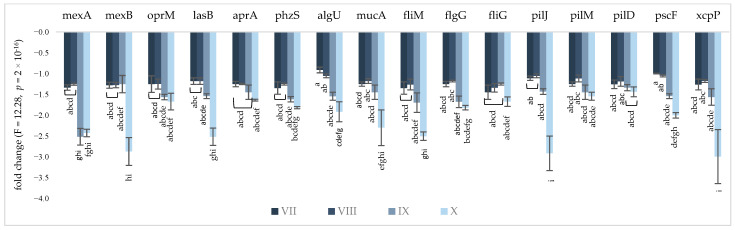
Impact of alternative NaCl compounds on the *P. aeruginosa* NT06 AMR and VF gene expression levels under in situ conditions. Values are presented as a fold change in expression in relation to control cultures and normalized to the non-differentially expressed 16S rRNA gene. Error bars indicate standard deviations from three replicates. Medium variants: VII—5.0 g/L KCl; VIII—6.0 g/L KCl; IX—KCl 6.0 g/L + NaL 6.0 g/L; X—KCl 6.0 g/L + NaL 6.0 g/L + NaC 2.5 g/L.

To emphasize the effect of KCl, NaL, and NaC on genes involved in the AMR and VF of *P. aeruginosa* NT06, changes in their expression were determined via RT–qPCR analysis. The transcriptional levels were normalized to the non-differentially expressed reference 16S rRNA gene. The fold change values of selected genes are presented in Figure 2 and Figure 3, for in vitro and in situ conditions, respectively. The results showed that the expression of all analyzed genes was considerably decreased due to the treatment, and the highest reduction in transcriptional levels was observed for the combination of KCl/NaL/NaC compounds (the fold change ranged from −1.44 to −3.40). The relative change in gene expression obtained in FJ medium was equal to that from cells cultivated in in vitro conditions. 

NaCl alternative compounds effectively decreased the expression of genes encoding the MexAB-OprM efflux pump, e.g., the addition of KCl/NaL resulted in 3.37-, 1.42-, and 2.01-fold decreases in the expression of mexA, mexB and oprM, respectively, and there were no statistically significant differences in treatment with KCl/NaL/NaC. The lowered expression of the phzS, aprA, and lasB genes corresponded with the spectrophotometric results regarding pyocyanin, elastase, and protease inhibition due to the NaCl alternative treatment. The expression of lasB was reduced by 2.00-fold and by 3.37-fold after treatment with KCl/NaL and KCl/NaL/NaC, respectively. The mRNA level of aprA was decreased, but not to the same extent; supplementation of mTSB medium with KCl/NaL and KCl/NaL/NaC resulted in aprA reduction by 1.57- and 1.67-fold, respectively, while in FJ medium the same compounds lowered the aprA gene by 1.45- and 1.64-fold, respectively. Other VFs tested (alginate-, flagella-, pili- and secretion-related genes) were also considerably inhibited by KCl/NaL and KCl/NaL/NaC. Treatment with only KCl at both concentrations was less effective at changing gene expression. 

## 3. Discussion

Given the adverse effects of excessive NaCl intake on health, the reduction in NaCl content within food products is now in high demand. In minimally processed fish-based products, which have recently gained more interest in the food market, NaCl should be replaced with alternative compounds that will exert equal preservation effects, due to the high risk of occurrence of foodborne pathogens, such as *P. aeruginosa*, that are inherently associated with the aquatic environment [1]. The high probability of this pathogen’s persistence in fish, as well as virulent and antimicrobial features, induced the need for research concerning the quality and safety of minimally processed fish-based products. This study aimed to characterize the antimicrobial potential of *P. aeruginosa* NT06 isolated from fish and to evaluate pyocyanin biosynthesis, elastase and protease activity upon treatment with compounds that can replace NaCl (KCl, NaL, and NaC) in minimally processed fish-based products. To fully reflect the native growth conditions of *P. aeruginosa* isolated from fish, mTSB medium with fish peptone and FJ medium were applied. 

Pseudomonad metabolic activity responsible for virulence and food spoilage may be more intense at lower temperatures than at the temperature considered optimal for cell growth [20,39,40]. Fish-based products have a high probability of containing potential reservoirs of antibiotic resistant *Pseudomonas* [18]. Therefore, considering the pathogenic nature of *P. aeruginosa*, along with the increased prevalence of antimicrobial resistance phenotypes [41], the antibiotic resistance profile was determined and served as a selection of examined strains. The present work revealed that the six examined *P. aeruginosa* strains isolated from commercially available fish had the ability to grow at 4 °C and were resistant to AMP, CRO and NA. The effective antibiotics for all analyzed strains included GEN, MEM, and CIP. Similar antibiotic resistance patterns for β-lactam (AMP), third-generation cephalosporins (e.g., CRO), and fluoroquinolones (e.g., NA) were established for *Pseudomonas* spp. isolated from fresh dairy products [21]. *P. aeruginosa* isolated from frozen meat and chicken nuggets also showed resistance to AMP and CRO [42]. In addition, the highest percentage of *P. aeruginosa* strains (89.65%) isolated from fresh and frozen meat and meat products were also resistant to AMP [43]. Resistance to AMP among pseudomonads is usually mediated by enzymes that degrade antibiotics that belong to the β-lactamase class. *P. aeruginosa* showed intermediate resistance towards TE. The above phenomenon is probably due to the occurrence of MexAB/MexXY efflux pump systems in *P. aeruginosa* cells [44]. Antimicrobial resistance of *P. psychrophila* isolated from fish was determined via efflux pump MexAB-OprM [45]. Furthermore, other RND family efflux pumps, such as MexCD-OprJ and MexEF-OprN, have been involved in the extrusion of β-lactams and quinolones, respectively [41]. Interestingly, the present study showed that one of the *P. aeruginosa* isolates (i.e., *P. aeruginosa* NT06) was resistant to NOR, an antibiotic from the fluoroquinolone class. Because resistance to fluoroquinolones is mainly attributed to overexpression of efflux pumps, it was hypothesized that the analyzed strain is characterized by the enhanced functioning of RND family proteins.

Therefore, to establish the transcriptome features of *P. aeruginosa* NTO6 grown under conditions that mimic the isolation source (mTSB medium), RNA-seq analyses were performed. The two-step approach was used: transcriptome data were mapped to the reference *P. aeruginosa* genome and were de novo assembled and then screened for the presence of genes classified into the AMR and VF groups according to bioinformatic tools such as the CARD and VFDB databases. Hence, the list of potential AMR and VF genes was extended with those that were not calculated in the reference-guide method, which, according to Raghavan and coauthors [46], might not be able to reconstruct all of the present transcripts. For instance, examples of gene products related to the predicted phenotype conferring the antibiotics inactivation through enzymatic reactions and related to the efflux of antibiotics, as well as HSI-1 secretion system apparatus were only identified in the *P. aeruginosa* NT06 transcriptome assembled de novo. Major identified AMR products concerned RND efflux pumps (mainly MexAB-OprM encoding genes), which are known for a lack of specific, effective extrusion of antimicrobials outside the cells; nevertheless, they were not always responsible for antibiotic resistance [16]. Antimicrobial efflux is also modulated by a two-component regulatory system, to which five gene products from the *P. aeruginosa* NT06 transcriptome were classified. Additionally, the two-component system has an essential role in the regulation of VF among *P. aeruginosa* [47]. RsmA protein is involved in the initial colonization of *P. aeruginosa* and the development of acute pneumonia [48]. *P. aeruginosa* NT06 was also characterized by the presence of the antibiotic resistance gene soxR, which is activated by pyocyanin [49]. The enhanced resistance of *P. aeruginosa* cells is also attributed to decreased membrane permeability to antimicrobials [41]. In addition to porins (opmH, oprN, opmE), our study recognized the AMR determinant arnA, in the *P. aeruginosa* NT06 transcriptome, which alters cell wall charge and thus has an additional effect on membrane-mediated resistance [50]. According to CARD analysis of the genomes of Pseudomonads isolated from the salmon processing environment, the major AMR elements detected were RND efflux pumps, soxR, and adeF gene products [29]. Our study also indicated a wide range of VFs among the *P. aeruginosa* NT06 transcriptome, and the most abundant were genes involved in alginate production and regulation. Alginate is one of the major constituents of exopolysaccharides, which contribute to decreased susceptibility to antimicrobials of biofilms [51]. Similar results were obtained by Poursina and coauthors, who showed the presence of algD VF among the *P. aeruginosa* strains obtained from raw meat [24]. 

In the food chain, especially in foods produced with minimal processing technology, bacteria encounter different sublethal stressors, which influence their response and resistance mechanisms [52]. Dietary recommendations for salt reduction have emerged from the need to search for compounds that exert similar preservative effects with no simultaneous adverse impact on health [5]. In this study, we hypothesized that NaCl replacement with combinations of different salts, KCl, NaL, and NaC, decreased the *P. aeruginosa* virulence phenotype, which is also perceived as significant in regard to fish spoilage processes, i.e., pyocyanin biosynthesis and elastase and protease activity. In fish products, the above pseudomonad traits result in changed organoleptic properties, mostly discoloration and degradation of tissue [33]. Spectrophotometric analyses showed the effectiveness of analyzed compounds for lowering the *P. aeruginosa* VF phenotype. The inhibitory effects of NaC on biofilm development, motility, pyocyanin production and proteolytic activity of *P. aeruginosa* were also confirmed in the work of Khayat and co-authors [53]. The antivirulence activity of low doses of NaC (5%) was also established for the fish-borne pathogen *Serratia marcescens*, which inhibited biofilm formation, diminished swarming motility and decreased protease activity [54]. The inhibitory effects of NaL on the foodborne pathogen *S. aureus* and staphylococcal enterotoxin were evaluated by Lin and coauthors [55]. Changes in the expression of genes involved in glycolysis, DNA repair, and cell division have been also reported after exposure of *Listeria monocytogenes* to NaL [56]. 

In the study, the confirmation of phenotype features was achieved via RT–qPCR analyses, which indicated lowered mRNA levels of selected genes encoding AMR and VF. Moreover, comparative transcriptomics showed the effect of 4% NaL on increasing the expression of virulence genes (actA, clpE, hly, ip, inlA, inlE, mpl, plcA and plcB) of *Listeria monocytogenes* [57]. Therefore, the use of organic acids as effective antimicrobial agents in food should be widely studied; these agents should be combined with other compounds (e.g., NaC) to ensure food quality and safety.

In conclusion, the following study provides insight into the potential AMR and VF among foodborne *P. aeruginosa* and the possible impairment of those features by KCl, NaL, and NaC, which exert synergistic effects and can be used in minimally processed fish-based products.

## 4. Materials and Methods

### 4.1. Microorganisms and Culture Conditions

Six *P. aeruginosa* strains isolated from commercially available raw salmon were used in this study. The bacterial cultures were grown in modified TSB medium (mTSB) (g/1000 mL of distilled water: 20.0 g of fish peptone; 2.5 g of glucose; 5.0 g of sodium chloride (NaCl); 2.5 g dipotassium phosphate (K_2_HPO_4_)) at 4 °C for 72 h. The tested cultures were supplemented with selected concentrations of KCl/NaL/NaC (Table 4). Reference cultures were grown in TSB medium (Oxoid, UK). For in situ analyses, *P. aeruginosa* was incubated in fish juice medium (FJ) obtained from fresh salmon fillets as described in our previous study [58].

### 4.2. Determination of Antibiotic Resistance

*P. aeruginosa* was analyzed for antibiotic resistance using the disc diffusion method as recommended by the Clinical and Laboratory Standards Institute [60]. In brief, aliquots of *P. aeruginosa* cultures were inoculated in sterile 0.85% NaCl to obtain a turbidity equivalent to 0.5 McFarland standard. Thereafter, the inoculum was plated on the Mueller Hinton agar plates (Thermo Fisher Scientific, Waltham, MA, USA) and standard discs with antibiotics were applied. After incubation at 37 °C for 24 h, the widths of the growth inhibition halos were measured. The following antibiotics (Oxoid, Thermo Fisher Scientific Australia Pty Ltd., Scoresby, Australia) were tested (μg/disc): AMP (10), GEN (10), CIP; 5), nalidixic acid (NA; 30), tetracycline (TE; 30), norfloxacin (NOR; 10), meropenem (MEM; 10), and ceftriaxone (CRO; 30).

### 4.3. Determination of the P. aeruginosa NT06 Genes Involved in AMR and VF

Initially, total RNA was isolated from *P. aeruginosa* NT06 cells cultured aerobically in mTSB medium (with 5 g/L NaCl) for 72 h at 4 °C using the RNAqueous Kit (Thermo Fisher Scientific, Waltham, MA, USA). The Ribominus Transcriptome Isolation Kit (Invitrogen, Thermo Fisher Scientific, Waltham, MA, USA) was used to remove the ribosomal RNA. Transcriptomic libraries were constructed using the Collibri™ Stranded RNA Library Prep Kit for Illumina™ and the Collibri™ H/M/R rRNA Depletion Kit (Thermo Fisher Scientific, Waltham, MA, USA). The obtained libraries were then analyzed on a Qubit fluorometer 4.0 (Invitrogen, Thermo Fisher Scientific, Waltham, MA, USA). Whole transcriptome sequencing was performed with a MiSeq Reagent Kit on a MiSeq Illumina sequencer. Data were analyzed using CLC Genomics Workbench 20.0.4 (Qiagen, Germantown, MD, USA) software by mapping the reads to the corresponding *P. aeruginosa* PA01 genome and through estimation of transcript abundance with TPM (transcripts per million) values. The transcriptome was assembled de novo and analyzed for AMR and VF genes using the Comprehensive Antibiotic Resistance Database (CARD) and the Virulence Factor Database (VFDB), respectively [61,62]. RNA-Seq data were deposited in the NCBI Short-Read Archive (SRA) under the number SRX19555927.

### 4.4. Determination of Changes in the P. aeruginosa NT06 Virulence Phenotype

#### 4.4.1. Assessment of Pyocyanin Content

The method of Huerta et al. [63] was used for pyocyanin content determination. Briefly, after centrifugation of bacterial cultures (3000 g/10 min), 2 mL of chloroform (Chempur, Piekary Śląskie, Poland) was added to the cell-free culture supernatants and the optical density of the chloroform layer was measured at a wavelength of 690 nm (OD_690_). The percent of inhibition of pyocyanin synthesis was calculated as follows:

Pyocyanin inhibition = 100 − [A/B × 100]

where A is the OD_690_ of the chloroform layer containing pyocyanin from *P. aeruginosa* culture grown in mTSB medium with selected concentrations of test substances, and B is the OD_690_ of the chloroform layer containing pyocyanin from the reference *P. aeruginosa* culture.

#### 4.4.2. Determination of Elastase Activity

To measure the elastase activity of *P. aeruginosa*, an assay based on the cleavage of the elastase-specific chromogenic peptide substrate N-succinyl-Ala-Ala-Ala-p-anilide (Sigma–Aldrich, St. Louis, MO, USA) was used [64]. An aliquot of 100 µL of reaction mixture contained the cell-free supernatant, 1 mM of chromogenic substrate, and buffer (50 mM Tris-HCl, 10 mM CaCl_2_. Then, 1 mM ZnCl_2_, and 150 mM NaCl, pH 8.0 were incubated at 37 °C for 2 h. Next, an absorbance at a wavelength of 405 nm (A_405_) of the samples was measured in a microplate reader (Thermo Fisher Scientific, Waltham, MA, USA). The results were expressed in U l^−1^. The percentage of inhibition of elastase activity was calculated as follows:

Elastase inhibition = 100 − [C/D × 100]

where C is the A_405_ of samples containing the supernatant of *P. aeruginosa* culture grown in mTSB medium with selected concentrations of test substances, and D is the A_405_ of the reference *P. aeruginosa* culture.

#### 4.4.3. Determination of Protease Activity

Proteolytic activity was assayed by measuring the release of α-amino groups with the trinitrobenzenesulfonic acid (TNBS) (Sigma–Aldrich, St. Louis, MO, USA) method [65]. The method is based on the reaction of free amino groups with TNBS reagent at pH 9.2 in the dark. Next, the absorbance at a wavelength of 420 nm (A_420_) of the samples was measured in a microplate reader (Thermo Fisher Scientific, Waltham, MA, USA). The results were expressed in U l^−1^. The percentage of inhibition of protease activity was calculated as follows:

Protease inhibition = 100 − [E/F × 100]

where E is the A_420_ of samples containing the supernatant of *P. aeruginosa* culture grown in mTSB medium with selected concentrations of test substances, and F is the A_420_ of the reference *P. aeruginosa* culture.

### 4.5. Determination of Changes in the Levels of AMR and VF Gene Expression

#### 4.5.1. RNA Extraction and cDNA Synthesis

*P. aeruginosa* NT06 was grown on mTSB medium and on FJ medium as described in Section 2.1 and then the cultures were treated with the RNAprotect^®^ Bacteria Reagent (Qiagen, Hilden, Germany). Total RNA was extracted and purified using the PureLink™ RNA Mini Kit (Thermo Fisher Scientific, Waltham, MA, USA) and the PureLink™ DNase Set (Invitrogen, Waltham, MA, USA). RNA extracts were analyzed on a Qubit Fluorometer 4 (Invitrogen, Waltham, MA, USA) using Qubit™ XR RNA and Qubit™ IQ RNA Assay Kits (Thermo Fisher Scientific, Waltham, MA, USA) and then reverse transcribed to cDNA with a High Capacity RNA-to-cDNA Kit (Life Technologies, Carlsbad, CA, USA) according to the manufacturer’s protocol.

#### 4.5.2. RT–qPCR Analyses

The resulting cDNA was amplified on a CFX96 system (Bio-Rad, Hercules, CA, USA) using GoTaq^®^ Master Mix (Promega, Walldorf, Germany) and gene-specific primers for AMR and VF-related genes (Table 5). The following cycling conditions were applied: initial denaturation at 95 °C for 2 min; 40 cycles of denaturation at 95 °C for 15 s, annealing at 52 °C and extension at 72 °C for 15 s; followed by a melting curve. The fold changes in relative gene expression levels were estimated using the 2^−∆∆ct^ method [66] with regard to the reference gene encoding the 16S rRNA ribosome subunit.

### 4.6. Statistical Analysis

R Studio Software v. 4.3.1 [67] was used to conduct the statistical analyses. Significant differences (*p* < 0.05) were established via one-way analysis of variance (ANOVA) followed by Tukey’s post hoc test. The experiments were performed in triplicate.

## Figures and Tables

**Table 1:** Growth characteristics of *P. aeruginosa* strains.

Feature		*Pseudomonas aeruginosa* Strain					
		NT01	NT02	NT03	NT04	NT05	NT06
Growth at 4 °C		48 h	72 h	72 h	72 h	72 h	24 h
Antibiotic resistance (zones of inhibition in mm)	TE (30 μg; F = 1.822, *p* = 0.223)	12 ^a^	12 ^a^	12 ^a^	10 ^a^	12 ^a^	12 ^a^
	GEN (10 μg, F = 19.16, *p* = 0.000024)	21 ^b^	26 ^a^	22 ^b^	18 ^c^	20 ^bc^	20 ^bc^
	MEM (10 μg; F = 9.908, *p* = 0.000613)	20 ^bc^	23 ^a^	20 ^bc^	20 ^bc^	19 ^bc^	22 ^ab^
	AMP (10 μg)	0	0	0	0	0	0
	CRO (30 μg)	0	0	0	0	0	0
	NA (30 μg)	0	0	0	0	0	0
	CIP (5 μg; F = 80.6, *p* = 8.25 × 10^−9^)	25 ^bc^	26 ^b^	20 ^e^	30 ^a^	23 ^de^	24 ^cd^
	NOR (10 μg; F = 80.55, *p* = 8.28 × 10^−9^)	26 ^a^	20 ^b^	22 ^b^	25 ^a^	26 ^a^	15 ^c^

Zones of inhibition are mean values calculated from three replicates. Statistical differences were calculated using one-way ANOVA and a post hoc Tukey’s test. Means with the same letter do not differ significantly.

**Table 2:** AMR genes according to the CARD database and the transcriptome abundance in *P. aeruginosa* NT06.

Antimicrobial Resistance Gene	Predicted Phenotype	Contig	Position in Contig	% Identity	Gene ARO	TPM Value
OXA-850	Antibiotic inactivation enzyme	1_S1_L001_(paired)_contig_1174	3524..4312	99.61	3005138	-
OXA-486		1_S1_L001_(paired)_contig_1174	3524..4312	99.61	3003643	-
fosA		1_S1_L001_(paired)_contig_1794	1354..1761	99.75	3000149	-
APH(3’)-IIb		1_S1_L001_(paired)_contig_84	3972..4778	98.63	3002645	-
soxR	Antibiotic-resistant gene variant/mutant	1_S1_L001_(paired)_contig_347	45..515	99.15	3004107	8.410823439
mexP	Efflux pump complex or subunit conferring antibiotic resistance	1_S1_L001_(paired)_contig_1116	439..1595	99.91	3003698	-
opmH		1_S1_L001_(paired)_contig_1295	1..1202	100	3003682	-
oprN		1_S1_L001_(paired)_contig_1828	213..1124	99.78	3000805	9.234260698
opmE		1_S1_L001_(paired)_contig_1932	391..1287	99.44	3003700	-
mexJ		1_S1_L001_(paired)_contig_2174	1..689	98.40	3003692	-
pmpM		1_S1_L001_(paired)_contig_2344	634..2067	99.86	3004077	-
triA		1_S1_L001_(paired)_contig_2475	148..1299	99.82	3003679	-
emrE		1_S1_L001_(paired)_contig_396	2109..2389	98.93	3004038	-
mexX		1_S1_L001_(paired)_contig_564	1059..1880	98.05	3003034	-
yajC		1_S1_L001_(paired)_contig_580	337..675	99.70	3005040	-
mexA		1_S1_L001_(paired)_contig_91	206..1357	99.82	3000377	50.25938765
mexB		1_S1_L001_(paired)_contig_91	1373..4513	99.39	3000378	77.32260128
oprM		1_S1_L001_(paired)_contig_91	4515..5972	99.58	3000379	106.8020152
arnA	Gene-altering cell wall charge	1_S1_L001_(paired)_contig_1042	2317..4314	98.99	3002985	15.62721041
rsmA	Protein(s) and two-component regulatory system modulating antibiotic efflux	1_S1_L001_(paired)_contig_33	1050..1235	99.46	3005069	90.10851165
armR		1_S1_L001_(paired)_contig_34	1046..1207	100	3004056	110.9821332
parS		1_S1_L001_(paired)_contig_361	3186..4443	98.96	3005067	9.234260698
basS		1_S1_L001_(paired)_contig_403	3425..4858	100	3003583	-
mexL		1_S1_L001_(paired)_contig_693	1087..1725	99.84	3003710	-

“-“ indicates not present in the reference-guided transcriptome. The TPM value is a normalized RNA-Seq results and indicates the number of transcripts that came from 1 million RNA molecules.

**Table 3:** VF genes according to the VFDB database and the transcriptome abundance in *P. aeruginosa* NT06.

Virulence Gene	Virulence Factor (ID)	Contig	Position in Contig	% Identity	TPM Value
algU	Alginate (VF0091)	1_S1_L001_(paired)_contig_290	1332..1913	100	419.3972733
mucA		1_S1_L001_(paired)_contig_290	1945..2529	99.82	308.3769521
mucB		1_S1_L001_(paired)_contig_290	2538..3488	99.89	101.2564359
mucC		1_S1_L001_(paired)_contig_290	3485..3940	99.78	52.12497157
algR		1_S1_L001_(paired)_contig_300	4786..5532	99.73	115.0388622
algZ		1_S1_L001_(paired)_contig_300	5537..6554	99.80	35.65093406
algD		1_S1_L001_(paired)_contig_3027	1..880	99.88	3.486620172
algL		1_S1_L001_(paired)_contig_3318	1..676	99.70	5.520481939
algQ		1_S1_L001_(paired)_contig_687	214..696	99.58	72.55490549
algB		1_S1_L001_(paired)_contig_871	292..1641	99.92	26.63571197
algC	Alginate biosynthesis (CVF522)	1_S1_L001_(paired)_contig_634	1481..2872	99.85	32.61213588
algW	Alginate regulation (CVF523)	1_S1_L001_(paired)_contig_107	2934..4103	99.91	19.27355951
mucP		1_S1_L001_(paired)_contig_132	9573..10925	99.70	-
mucD		1_S1_L001_(paired)_contig_290	3980..5404	99.78	88.53225941
mucE		1_S1_L001_(paired)_contig_3047	1..252	99.60	-
aprA	Alkaline protease (VF0090)	1_S1_L001_(paired)_contig_1207	1..1216	99.58	10.15768677
motB	Deoxyhexose linking sugar, 209 Da capping structure (AI138)	1_S1_L001_(paired)_contig_203	4505..5548	98.94	28.89686753
motA		1_S1_L001_(paired)_contig_203	5568..6419	98.12	15.73726119
motC		1_S1_L001_(paired)_contig_216	4870..5610	99.86	21.7958461
motD		1_S1_L001_(paired)_contig_216	5623..6513	100	17.44249243
flgN		1_S1_L001_(paired)_contig_325	4531..5001	99.57	-
flgM		1_S1_L001_(paired)_contig_325	5056..5379	100	67.71791179
fliK		1_S1_L001_(paired)_contig_336	149..954	100	-
fliL		1_S1_L001_(paired)_contig_336	1198..1719	99.80	-
fliA		1_S1_L001_(paired)_contig_632	2977..3720	99.86	-
motY		1_S1_L001_(paired)_contig_67	198..1163	99.89	-
fleS		1_S1_L001_(paired)_contig_88	1782..2990	99.00	17.39157288
flgF	Flagella (VF0273)	1_S1_L001_(paired)_contig_1247	41..790	100	8.532456885
flgG		1_S1_L001_(paired)_contig_1247	837..1620	99.61	9.304751238
flgH		1_S1_L001_(paired)_contig_1247	1669..2364	99.71	7.443132546
flgI		1_S1_L001_(paired)_contig_1247	2376..3485	99.45	23.88429051
flgJ		1_S1_L001_(paired)_contig_1247	3496..4698	99.58	9.119120291
flhA		1_S1_L001_(paired)_contig_1444	121..1682	99.87	12.33843308
fliM		1_S1_L001_(paired)_contig_336	1727..2698	99.79	22.88614611
fliN		1_S1_L001_(paired)_contig_336	2726..3199	100	21.21542173
fliO		1_S1_L001_(paired)_contig_336	3201..3653	98.67	18.16275117
fliP		1_S1_L001_(paired)_contig_336	3650..4417	99.47	8.332477427
fliQ		1_S1_L001_(paired)_contig_336	4462..4731	100	7.900423042
fliR		1_S1_L001_(paired)_contig_336	4731..5507	99.22	5.882830175
flhB		1_S1_L001_(paired)_contig_336	5510..6646	99.64	7.772372461
flgE		1_S1_L001_(paired)_contig_462	1..971	98.97	6.801042976
flgD		1_S1_L001_(paired)_contig_462	999..1712	99.71	14.08418754
fliE		1_S1_L001_(paired)_contig_618	211..540	99.69	11.08111284
fliF		1_S1_L001_(paired)_contig_618	563..2359	99.55	12.039996
fliG		1_S1_L001_(paired)_contig_618	2365..3381	99.80	23.37166867
fliH		1_S1_L001_(paired)_contig_618	3383..4189	99.13	-
fliI		1_S1_L001_(paired)_contig_618	4179..5534	99.04	14.60729292
fliJ		1_S1_L001_(paired)_contig_618	5548..5991	99.32	18.53091505
flhF		1_S1_L001_(paired)_contig_632	710..1999	99.68	26.69345593
fleN		1_S1_L001_(paired)_contig_632	2138..2980	99.88	21.32752738
fleQ		1_S1_L001_(paired)_contig_88	197..1669	99.11	38.27234322
fleR		1_S1_L001_(paired)_contig_88	2995..3864	99.19	13.50072292
tse1	HSI-1 (SS178)	1_S1_L001_(paired)_contig_1406	164..628	99.35	-
tse3		1_S1_L001_(paired)_contig_1773	1196..2241	99.80	-
tse2		1_S1_L001_(paired)_contig_2757	1..460	98.91	-
tagQ		1_S1_L001_(paired)_contig_397	766..1680	99.67	-
hsiA1	HSI-I (VF0334)	1_S1_L001_(paired)_contig_1341	329..1340	99.01	-
tagT		1_S1_L001_(paired)_contig_1644	142..861	98.47	-
tagS		1_S1_L001_(paired)_contig_1644	861..2060	98.83	-
tagF/pppB		1_S1_L001_(paired)_contig_1824	155..640	98.55	-
pppA		1_S1_L001_(paired)_contig_2001	1206..1712	99.80	5.434153415
hsiH1		1_S1_L001_(paired)_contig_354	932..1978	99.90	-
clpV1		1_S1_L001_(paired)_contig_354	1971..3922	99.53	7.649199338
hsiE1		1_S1_L001_(paired)_contig_652	292..1137	99.76	-
hcp1		1_S1_L001_(paired)_contig_652	1305..1793	100	58.57807093
hsiC1/vipB		1_S1_L001_(paired)_contig_652	1869..3365	99.79	-
hsiB1/vipA		1_S1_L001_(paired)_contig_652	3378..3706	100	-
waaF	LPS (VF0085)	1_S1_L001_(paired)_contig_106	1..970	99.17	16.44018668
waaC		1_S1_L001_(paired)_contig_106	967..2034	99.53	22.25560584
waaG		1_S1_L001_(paired)_contig_106	2031..3152	99.55	11.95021973
waaP		1_S1_L001_(paired)_contig_106	3149..3955	99.38	10.57305686
waaA		1_S1_L001_(paired)_contig_3715	1..1172	100	8.583960649
lasA	LasA (VF0088)	1_S1_L001_(paired)_contig_648	1331..2587	99.28	4.363684053
lasB	LasB (VF0087)	1_S1_L001_(paired)_contig_29	124..1620	99.73	82.23858626
phzC1	Phenazines biosynthesis (CVF536)	1_S1_L001_(paired)_contig_3114	24..930	99.44	0.250189329
pchC	Pyochelin (VF0095)	1_S1_L001_(paired)_contig_1475	1..676	99.85	6.85240774
pchD		1_S1_L001_(paired)_contig_1475	673..2000	99.62	7.229010656
pchA		1_S1_L001_(paired)_contig_1810	134..1564	99.51	12.56401508
pchB		1_S1_L001_(paired)_contig_1810	1561..1866	99.67	1.991703288
pchG		1_S1_L001_(paired)_contig_2287	1012..2061	99.33	9.577247524
fptA		1_S1_L001_(paired)_contig_2959	367..1908	99.54	5.353565842
phzS	Pyocyanin (VF0100)	1_S1_L001_(paired)_contig_4777	1..874	98.97	3.276673151
pvdH	Pyoverdine (IA001)	1_S1_L001_(paired)_contig_1063	148..1557	99.57	13.83174368
mbtH-like		1_S1_L001_(paired)_contig_1063	1635..1853	100	-
pvcD		1_S1_L001_(paired)_contig_4738	1..435	98.16	4.232369487
pvcA		1_S1_L001_(paired)_contig_4942	1..654	99.38	3.087442787
lasI	Quorum sensing (VF0093)	1_S1_L001_(paired)_contig_420	22..627	100	93.02831941
rhlI		1_S1_L001_(paired)_contig_950	202..807	98.67	13.0742503
rhlA	Rhamnolipid (VF0089)	1_S1_L001_(paired)_contig_106	4860..5747	99.43	6.176971683
rhlB		1_S1_L001_(paired)_contig_106	5813..7093	98.98	6.898663145
rhlC	Rhamnolipid biosynthesis CVF524)	1_S1_L001_(paired)_contig_1794	380..1357	99.69	6.543295157
pscB	TTSS (VF0083)	1_S1_L001_(paired)_contig_1578	342..764	98.58	2.881613268
exsD		1_S1_L001_(paired)_contig_1578	798..1628	99.75	7.334069869
pcrG		1_S1_L001_(paired)_contig_2551	216..512	99.66	2.052057933
pscG		1_S1_L001_(paired)_contig_3200	376..723	100	2.626987957
pscF		1_S1_L001_(paired)_contig_3200	726..983	100	4.724505474
pscE		1_S1_L001_(paired)_contig_3200	986..1189	99.01	1.493777466
pscK		1_S1_L001_(paired)_contig_3270	1..429	99.53	4.374123489
popB		1_S1_L001_(paired)_contig_491	164..1336	99.57	8.313196332
popD		1_S1_L001_(paired)_contig_491	1348..2235	99.54	5.490641496
exsC		1_S1_L001_(paired)_contig_743	101..538	100	11.13171153
exsE		1_S1_L001_(paired)_contig_743	547..792	99.59	-
exsB		1_S1_L001_(paired)_contig_743	801..1214	99.75	11.77702814
pcr2		1_S1_L001_(paired)_contig_793	170..541	98.92	-
pilU	Type IV pili (VF0082)	1_S1_L001_(paired)_contig_1243	1002..2150	100	15.64761147
pilT		1_S1_L001_(paired)_contig_1243	2328..3362	99.90	22.96520487
fimT		1_S1_L001_(paired)_contig_1412	1..435	99.31	1.792532959
pilF		1_S1_L001_(paired)_contig_230	6676..7434	99.73	28.10427169
pilY2		1_S1_L001_(paired)_contig_2608	185..532	100	3.50265061
pilE		1_S1_L001_(paired)_contig_2608	529..846	100	12.87594097
fimV		1_S1_L001_(paired)_contig_264	363..3107	98.15	32.68125308
pilM		1_S1_L001_(paired)_contig_359	910..1974	99.62	48.64244368
pilN		1_S1_L001_(paired)_contig_359	1974..2570	100	18.37571476
pilO		1_S1_L001_(paired)_contig_359	2567..3190	99.83	20.99906399
pilP		1_S1_L001_(paired)_contig_359	3187..3711	100	19.15449505
fimU		1_S1_L001_(paired)_contig_4477	1..442	100	4.808372435
pilG		1_S1_L001_(paired)_contig_49	1880..2287	100	87.38598175
pilH		1_S1_L001_(paired)_contig_49	2334..2699	100	50.78843384
pilI		1_S1_L001_(paired)_contig_49	2750..3286	99.81	41.99267156
pilJ		1_S1_L001_(paired)_contig_49	3371..5419	99.95	38.66762166
pilK		1_S1_L001_(paired)_contig_49	5480..6355	99.77	19.13262919
xcpA/pilD		1_S1_L001_(paired)_contig_524	1509..2381	99.31	12.21714903
pilX		1_S1_L001_(paired)_contig_539	2482..3069	99.82	6.218991899
pilW		1_S1_L001_(paired)_contig_539	3066..3565	99.4	6.648667703
chpA		1_S1_L001_(paired)_contig_613	28..5322	99.79	16.14221957
chpB		1_S1_L001_(paired)_contig_613	5315..6346	99.22	14.17351642
chpC		1_S1_L001_(paired)_contig_613	6343..6849	98.81	10.21779142
xcpT	xcp secretion system (VF0084)	1_S1_L001_(paired)_contig_4	714..1160	99.55	14.31620283
xcpU		1_S1_L001_(paired)_contig_4	1167..1685	99.80	20.55023335
xcpV		1_S1_L001_(paired)_contig_4	1682..2071	99.48	10.15768677
xcpW		1_S1_L001_(paired)_contig_4	2068..2781	99.57	7.255490549
xcpX		1_S1_L001_(paired)_contig_4	2778..3779	99.60	13.68550612
xcpY		1_S1_L001_(paired)_contig_4	3776..4752	99.89	20.42145904
xcpP		1_S1_L001_(paired)_contig_437	9180..9887	99.57	25.39421692

“-“ indicates not present in the reference-guided transcriptome. The TPM value is a normalized RNA-Seq result and indicates the number of transcripts that came from 1 million RNA molecules.

**Table 4:** Medium variants used in the study.

Designation	Medium Composition
I (reference mTSB)	mTSB + 5.0 g/L NaCl
II	mTSB + 5.0 g/L KCl
III	mTSB + 6.0 g/L KCl
IV	mTSB + KCl 6.0 g/L + NaL 6.0 g/L
V	mTSB + KCl 6.0 g/L + NaL 6.0 g/L + NaC 2.5 g/L
VI (reference FJ)	FJ + 5.0 g/L NaCl
VII	FJ + 5.0 g/L KCl
VIII	FJ + 6.0 g/L KCl
IX	FJ + KCl 6.0 g/L + NaL 6.0 g/L
X	FJ + KCl 6.0 g/L + NaL 6.0 g/L + NaC 2.5 g/L

mTSB—modified TSB medium, where casein-soybean peptone was replaced with fish peptone, FJ—fish juice medium prepared according to Dalagaard [59].

**Table 5:** List of genes evaluated in the RT–qPCR experiments.

Gene Name	Gene Definition, Coding Product and Role	Sequence (5′-3′) Fwd Rev	Tm (°C)	Size (bp)
16S rRNA	The small subunit ribosomal RNA, internal reference gene	GGAGACTGCCGGTGACAAACT TGTAGCCCAGGCCGTAAGG	56	75
mexA	RND multidrug efflux membrane fusion protein MexA precursor	AGCCATGCGTGTACTGGTTC CTCGGTATTCAGGGTCACCG	60	145
mexB	RND multidrug efflux transporter MexB	TGATAGGCCCATTTTCGCGT ATCCCGTTCATCTGCTGCTC	60	198
oprM	Major intrinsic multiple antibiotic resistance efflux outer membrane protein OprM precursor	GGTTCGGGTTCCTGGTTGTT GCAACTGCTCGGTGAAGGTA	60	193
lasB	Metalloproteinase (elastase), pseudolysin precursor	TGAACGACGCGCATTTCTTC CCCGTAGTGCACCTTCATGT	59	104
aprA	Alkaline metalloproteinase precursor	ATTGGTCAATGGCCATCCGT TGAACTTGCCCAGCGAGTAG	60	191
phzS	Probable FAD-dependent monooxygenase, involved in pyocyanin biosynthesis	CTGCAGTACCCGATGGTAGAC TTCTTCGTATTCGCGCAGGG	60	198
algU	Sigma factor AlgU, positive regulation of alginate biosynthesis	CGCGAGTTCGAAGGTTTGAG GCTTCTCGCAACAAAGGCTG	60	131
mucA	Anti-sigma factor MucA, involved in alginate regulation	GTGAAGCCCTGCAGGAAACT GCAGCGATATCCAGCTTCGG	60	181
fliM	Flagellar motor switch protein FliM	CGAGTACGTCAACTCGGAGG TAGGGCATGGTGATGTGCAG	60	129
flgG	Flagellar basal-body rod protein FlgG	CAACCTGGCCAACGTATCCA ACACCGGTACCCAATTGCAG	60	144
fliG	Flagellar motor switch protein FliG	CGAAGGCCAGCTGATGGATT GTACGTCCGAGGAGACTTCC	60	146
pilJ	Twitching motility protein PilJ	ACACCCAGTCGAACCATGAC ACCAGGATGTTCCAGCGTTT	60	169
pilM	Type 4 fimbrial biogenesis protein PilM	TCCTTGAACGGACGCAGAAC CGGACGCACCATCTATACCC	60	162
pilD	Prepilin leader peptidase/N-methyltransferase, role in type IV pili and type II pseudopili formation	CTGATCGCCAACCATTTCGG ACCAGCTTGAACAGCCAGAA	59	107
pscF	Type III export protein PscF,	GCGCAGATATTCAACCCCAAC TGATCTTGTGTTGCAGCTCG	60	169
xcpP	Secretion protein XcpP	CCCTCGGCGATCTTCAGACA GGCGATGATCAGGGCAACAG	61	124

## Data Availability

The data presented in this study are available on request from the corresponding authors.
